# Scope and Policy

**Published:** 1912-04

**Authors:** 


### Scope and Policy.

We had something to say on this subject in the September 1911 issue, at the formal beginning of the present editorial management. Not much remains to be said except on a few points in regard to which it is well to have a perfect understanding. It is obvious that the Buffalo Medical Journal cannot exist solely as a municipal organ. On the other hand, certain rather arbitrary and let us hope not immovable prejudices render it impracticable, at least for the present, to expect a general circulation such as might be achieved from New York, Boston and a few other points. Hence, a reasonable compromise is to consider this journal as a local medium, but for western New York and adjacent states, and not for one city or even county.

In all departments of the JOURNAL, we are endeavoring to carry out this policy as equably as possible. If, on the one hand, any of our Buffalo friends feel that more space should be devoted to purely local matters, we would ask them to consider, first, that it is impossible to carry on a creditable journal with a support thus restricted in numbers; secondly, that many things which it is theoretically desirable to record, do not make interesting reading except to a comparatively few persons; thirdly, that in any case, it is necessary to remember the very large number of organizations and interests in our general territory, all of which should have an equable representation. If on the other hand, our subscribers in other cities and towns feel that too much attention is paid to Buffalo, we ask them to remember that Buffalo, has a greater medical population than any other sectional unit in our territory; also the very practical point that we are dependent in the original department largely upon voluntary contributions and, in the editorial and news departments largely upon the length of our tether which, for a man engaged in medical practice, is not very long. So, if any one, anywhere feels that his own section is being slighted, let him give us the material to correct the omission.

Secondly, with regard not to geographic but literary and scientific scope, it is obvious that, without increasing the size of the journal considerably, the editor must exercise the selective activity of secretory cells. With the exception of the very valuable historic series of the late Lt. Col. Potter, or at least until that series shall have been completed, early in the next volume, it has been decided that the original department must be strictly limited to practical, technical articles. This is no mere personal opinion but the general view of those whom it has been possible to consult. We, therefore, ask that no one be offended if congratulatory, historic and other addresses or articles dealing with purely ethical matters, are not considered available.

Conversely, it seems best that the editor should keep out of the original department and publish his own practical medical articles in other journals. Even regarding matters of policy and polity, it has been and will be our endeavor to secure collaborators and, otherwise, to avoid purely personal views so far as possible, at any rate to the extent of leaving an opportunity for everyone, in every department of the JOURNAL from cover to cover, to express himself as he pleases, provided that ordinary accepted rules of debate and of ethics are followed.

---

Just a few words about our standing offer to publish *full* transactions of any professional organization in our territory at cost. We are, as stated, very glad to publish brief news notes of anything of interest in our territory. A few critics have expressed the opinion that we ought to publish transactions of a few societies free, as a matter of professional interest. Remembering that there are about 35 county societies, about ten large municipal or analogous regional societies, at least a hundred hospital and restricted organizations, in western New York, the impossibility of this plan becomes evident. It is obvious that there is no graft in charging printers' rates for pages added to the journal. In fact, if any considerable number of organizations accepted the offer, the incidental expenses of binding, postage, etc., would become so great that the offer would have to be withdrawn. But, for the present, it is open to any society that wishes to provide thus for preserving its records. Let it be understood that space thus taken will not be subtracted from the present number of pages of the JOURNAL but that extra pages will be added to compensate.

If anyone feels aggrieved that the minutes or reports or records of his particular institution are not considered available, let him ask himself if the same general kind of material would interest him if it referred to a society, or other organization of an entirely different nature and in a different city or town.

